# Oxygen, Ulcers, and Micrococci

**Published:** 1897-02-13

**Authors:** 


### MEDICAL PROGRESS AND HOSPITAL CLINICS.

[*The Editor will be glad to receive offers of co-operation and contributions from members of the profession. All letters should be addressed to* THE EDITOR, AT THE OFFICE, 28 & 29, SOUTHAMPTON STREET, STRAND, LONDON, W.C.]

### OXYGEN, ULCERS, AND MICROCOCCI.

It seems not altogether to the credit of London surgery that so little notice should have been taken at the great metropolitan hospitals of the suggestions put forward, and the experiments made by Dr. George Stoker, in regard to the effect of oxygen in promoting the healing of wounds. He has again and again shown his cases, and described the processes which he adopts. Within the last week he has done so twice, on February 4th, before the Harveian Society, and on February 8th, before the Medical Society, and it seems not unfair to urge that if there is nothing in this treatment it is time someone got up and said so; while if it really is of value some greater recognition should be given to it than has hitherto been the case.

At the Medical Society both his methods and his results were freely enough criticised, but there was no sign that any of the speakers had attempted to prove or disprove the statements put forward as to the effect of oxygen.

The points raised by Dr. Stoker are perfectly clear and definite, and ought to be capable of immediate proof or refutation. Describing his methods and results at the last meeting of the Harveian Society, he summarised them to the following effect: (1) By mere cleanliness and exposure to an atmosphere consisting of equal parts of oxygen and washed air, in other words to clean air containing three times its normal proportion of oxygen, wounds can be made to heal quickly which have resisted many other forms of treatment, although carried out for considerable lengths of time and by thoroughly competent surgeons. (2) This is not due to the cleanliness and moisture of the air alone. The oxygen is essential. If oxygen is not used the effect is not produced, while its activity is shown by the fact that, if it is used in too large a proportion, the wound becomes inflamed. (3) Among the events which follow and accompany the use of the oxygen is an alteration in the character of the bacteria present in the discharges from the wound. On coming under treatment these are usually of very diverse forms, and by ordinary bacteriological methods cultures of many different organisms can be obtained from them; but as the treatment proceeds, and as the wound takes on the healing character, the cultivations from the discharges consist more and more of the staphylococcus pyogenes albus or aureus, to the exclusion of other microbes, until at last they become pure cultures of these organisms. (4) In some cases, where but slow progress is being made, the healing is hastened by sowing the wound with artificially-prepared pure cultures of staphylococcus pyogenes aureus; and in certain cases which had been brought to an aseptic or sterile condition, and yet refused to heal even under oxygen, sowing with the staphylococcus was at once followed by cicatrisation. Surely these results are sufficiently curious, and are put forward with sufficient formality and definitiveness to deserve investigation and either confirmation or refutation.

If it were a mere matter of healing chronic ulcers we should probably not refer to it in this manner. It is possible that Dr. Stoker has a somewhat exaggerated idea of the difficulty of what most surgeons will look upon as a comparatively simple feat. The interest of the matter seems to us to lie chiefly in the bacteriology, and it is by that that the whole proceeding must stand or fall.

The staphylococcus pyogenes aureus is known to surgeons as the active factor in the production of that intensely septic and virulent disease osteomyelitis, a disease very apt to be followed by septic absorption, pyæmia, and death, unless free vent is quickly given to the discharges. To physicians it is known as one of the commonest causes of infective endocarditis with all its serious consequences. To those who have worked at the processes of wound infection, and have sought for the best means of disinfecting the skin, it and the S. pyogenes albus are known as almost normal inhabitants of the human integument, which are very difficult to get rid of, and which, especially the latter, are a very frequent cause of infection of operation wounds; producing deep suppuration if the infection take place at the time of the operation, and a superficial infection of the surface if they only gain access to it after the deeper parts have united, but in any case preventing the proper healing of the wound. To Dr. Stoker, however, these microbes appear as friendly and beneficent organisms, gifted with considerable efficacy in promoting the healing of wounds. Is this an error of observation? Is it from some laxity in the bacteriological methods that such a result has been obtained, or is it due to the fact that the micro-organism when growing in different circumstances, in fluids more highly charged with oxygen sets free different products? Who can answer until he has tried? Who can decide until he has investigated? In regard to this it is not, perhaps, irrelevant to point out how different the toxic effects of the same organism seem to be, according as it grows within the tissues or on the surface, where it presumably obtains a larger supply of oxygen; which would fall in with Dr. Stoker's statement that when more oxygen still is supplied it becomes harmless. Why it should become actually beneficent, we confess is quite beyond the range of our imagination. But facts put forward so repeatedly, and with such confidence that an institution has been established for the furtherance of the cult, should not be left in doubt. They should be either proved or disproved.

We repeat, then, our opinion that the time has come when these matters should no longer be left in the hands of one investigator. It is not a mere question of how to heal ulcers, but one touching the much larger question of how so to modify the conditions of growth of pathogenic organisms as to modify their products and, perhaps, lessen their virulence. If the statements made can be maintained, their applications go far beyond ulcers; if they are but idle dreams, let it so be said.

